# Insights from the high-altitude animal gut adaptation model: mechanisms of obesity regulation via microbiota-derived metabolite homeostasis and the gut-X axis

**DOI:** 10.3389/fmicb.2026.1795452

**Published:** 2026-03-04

**Authors:** Lijuan Cao, Wanlong Zhu

**Affiliations:** 1 Key Laboratory of Ecological Adaptive Evolution and Conservation on Animals-Plants in Southwest, Mountain Ecosystem of Yunnan Province Higher Institutes College, Yunnan Normal University, Kunming, China; 2 School of Life Sciences, Yunnan Normal University, Kunming, China

**Keywords:** gut microbiota, gut-X axis, high-altitude animals, obesity intervention, secondary bile acids, short-chain fatty acids

## Abstract

The unique environmental conditions at high altitudes drive the gut microbiota of resident animals to develop distinct structural and functional traits, thereby offering an ideal natural model for investigating the synergistic adaptation of hosts and microorganisms to extreme environmental stressors. This review systematically expounds the mechanism of metabolic adaptation of gut microbiota to high-altitude through the phenotypic characteristics of “high productivity and low inflammation,” and understands the mediating effect of short-chain fatty acids and secondary bile acids, which are derived metabolites of flora. SCFAs can enhance the intestinal barrier, regulate the function of immune cells, act on the gut-brain axis, and then affect the feeding behavior. SBAs, as signal molecules, regulate the lipid and energy metabolism of the host through the gut-liver axis. This division of labor and coordination, driven by different metabolites and achieved through specific gut-X axis pathways, constitutes a microecological regulatory network that enables the host to maintain metabolic homeostasis in high-altitude areas. Understanding this natural model can reveal the role of “flora metabolite organ axis” in maintaining health. It can also provide reference direction for obesity intervention caused by high-fat diet (HFD) and other factors, such as regulating the function of gut microbiota through strategies such as dietary regulation, probiotics and prebiotics supplementation, and fecal microbiota transplantation (FMT), and regulating the specific gut–X axis pathway, so as to restore metabolic balance.

## 1 Introduction

The global prevalence of overweight and obesity has risen steadily, affecting approximately one-third of the world’s population (Lin and Li, 2021). Accumulating research has demonstrated that obesity markedly elevates the risk of metabolic disorders, including Type 2 diabetes and cardiovascular diseases (Falahee et al., 2025; Jastreboff et al., 2019; Mechanick et al., 2012). High-fat diet (HFD), as a key inducement, can cause obesity by changing the composition of gut microbiota and reducing the diversity of gut microbiota (Boonpor et al., 2022; Cao et al., 2024; Ma et al., 2025; Qiao et al., 2025; Sonnenburg and Bäckhed, 2016; Tao et al., 2024). However, nature provides a reverse model for the “food fat gut microbiota host metabolism” chain of obesity, that is, high-altitude adaptive animals. Due to lower dietary fat content at high altitudes, the gut microbiota of high-altitude–adapted animals has evolved adaptive features suited to a low-fat diet (Zhang A. et al., 2022). Through modulation of the composition and diversity of the flora, high-altitude adaptive animals can efficiently use low-fat and high-fiber diet, maintain their own energy homeostasis, and resist flora imbalance and excessive energy accumulation caused by exogenous HFD.

Gut microbiota is an important “microbial organ” of the host, which can decompose indigestible dietary components and produce active metabolites, affecting the way of nutrition absorption, energy distribution and fat storage of the host (Agus et al., 2021; Ren et al., 2025a,b). The gut plays a vital role—not only as the primary site for digestion and nutrient absorption, but also as an essential organ involved in metabolism and immune regulation. As such, imbalances in the intestinal microbiota are closely linked to a broad spectrum of health conditions (Fitzgibbon and Mills, 2020; Torres-Fuentes et al., 2017). Studies have shown that the imbalance of gut microbiota can lead to the occurrence and development of obesity through multiple mechanisms (Clavel et al., 2014; Islam et al., 2023). The gut microbiota in high-altitude-dwelling animals optimized the production of core flora-derived metabolites, regulated the function of organs, and enhanced the metabolic ability to cope with extreme environmental pressure. Among the many flora-derived metabolites, short-chain fatty acids (SCFAs) and secondary bile acids (SBAs) play a crucial role as the mediators of the “flora metabolite gut-X axis” regulatory network.

Short-chain fatty acids are mainly produced by gut microbial fermentation of indigestible dietary components (e.g., fiber) and critically regulate local/systemic immunity, inflammation, and energy metabolism. The changes of SCFAs are closely related to chronic diseases such as obesity. Studies have shown that the intestines of white-lipped deer (*Przewalskium albirostris*) in the cold environment in winter can specifically enrich SCFAs producing bacteria, significantly improve the levels of butyric acid, valeric acid and other metabolites, and enhance the ability to cope with environmental pressure by enhancing carbohydrate metabolism and energy conversion (Li et al., 2025). In the yak (*Bos grunniens*), the transcription factor HNF4A regulates the transport function of SCFAs in intestinal epithelial cells. Concurrently, an energy supply network is established in collaboration with microorganisms such as *Bacillus* (Huang et al., 2025; Kulyar et al., 2025). The “high productivity, low inflammation” SCFAs regulation mode of animals adapted to high-altitude is in sharp contrast to the decline of SCFAs level and inflammatory disorder under obesity (Komisarska et al., 2024; Vijay and Valdes, 2022). Bile acids (BAs) metabolism is the core of the connection between flora and host lipid metabolism. Obese individuals are often accompanied by metabolic disorders of BAs, including synthetic changes, abnormal intestinal and hepatic circulation, and dysfunction of nuclear receptor signal transduction (Manothiya et al., 2025). Studies have shown that sterol regulatory element-binding transcription factor 2 (SREBF2), an active transcription factor in the gut of yak, can target and regulate the BAs transporter gene *SLC10A2*, so as to optimize the efficiency of enterohepatic circulation. However, the disorder of this pathway is the core link of the metabolic disorder of BAs in obese individuals. It is speculated that the flora of animals at high-altitude can weaken the metabolic abnormalities caused by the signal disorder of this nuclear receptor (Gou et al., 2023; Trabelsi et al., 2017; Ye et al., 2024). As the main metabolites produced by the gut microbiota, SCFAs and SBAs - a microbiota derived subtype of BAs - play different but complementary roles in host metabolism. Specifically, SCFAs regulate appetite signals through the gut-brain axis and balance immune responses through the gut-bone marrow axis, while SBAs, as a key functional subtype mediating microbiota host communication, regulate lipid metabolism through the gut-liver axis. The two flora-derived metabolites together constitute a two-way communication pathway between flora and distal organs, which not only ensures the metabolic homeostasis of high-altitude animals in extreme environments, but also provides a natural reference for further understanding the pathological causes of obesity caused by HFD.

At present, methods such as “medicine and food homology (MFH)” (Liu et al., 2025), supplementation of specific dietary ingredients or prebiotics and probiotics have shown unique potential in regulating intestinal microecology and become the frontier direction of obesity intervention. These methods can promote the growth of beneficial bacteria, increase the production of SCFAs, and maintain BAs homeostasis (Gong et al., 2020; Ng et al., 2023; Qu et al., 2023). This review systematically summarizes advances in this field and explores microecological intervention strategies inspired by high-altitude animal models, thereby laying the groundwork for the development of novel strategies against obesity.

## 2 Mechanisms driving obesity

### 2.1 Effect of high-fat food on gut microbiota structure

Metagenome sequencing and 16S rRNA gene analysis technology have brought the study of gut microbiota into a new stage (Gomes et al., 2018). Trillions of microorganisms are colonized in the gut, forming a complex microecology. The stability of the gut microbiota is intimately associated with the physiological health of the host (Abbott, 2016; Liu et al., 2021; Sender et al., 2016). The gut microbiota of healthy individuals mainly depends on the indigestible polysaccharides in the diet, intestinal mucus and intestinal epithelial cells (Turnbaugh et al., 2009). By fermenting these substrates, the flora can produce active metabolites such as SCFAs, which can further regulate the physiological function of the host (Patterson et al., 2016). From the perspective of phylum classification, Firmicutes and Bacteroidetes predominate in the gut microbiota of healthy adults, acting as the core microbial phyla (Reyes et al., 2010). Although the composition of gut microbiota varies among individuals, the core functions of metabolism, fermentation and lipopolysaccharide (LPS) biosynthesis are relatively conservative (Ahrodia et al., 2022; Qin et al., 2010).

Food is a key external factor in shaping the structure of animal gut microbiota. Among them, HFD is related to animal obesity and the disorder of specific flora. Early studies focused on the ratio of Firmicutes/Bacteroides (F/B) ratio, and believed that it was positively correlated with the increase of host energy acquisition and obesity (Indiani et al., 2018; Karačić et al., 2024; Ley et al., 2005). The new research points out that the relationship between F/B value and obesity is affected by many factors, such as taxonomic resolution, individual differences and food structure, and its indicating significance tends to be complex (Koliada et al., 2017). In obese individuals, the abundance of the microbiota that promote obesity increases, such as Firmicutes phylum, Proteobacteria, Enterobacteriaceae, and specific *Bacteroides* species (Bibbò et al., 2016; Kim et al., 2013; Wu et al., 2011). While protective microbiota such as *Akkermansia muciniphila*, *Christensenellaceae*, *Bifidobacterium*, and certain *Lactobacillus* species show reduced abundance (Million et al., 2012; Thingholm et al., 2019). These protective bacteria exert anti-obesity effects through multiple mechanisms, including strengthening the intestinal barrier, regulating lipogenesis, reducing inflammation, promoting white adipose tissue (WAT) browning, and improving insulin sensitivity (Chen et al., 2018; Hsieh et al., 2016).

The natural low-fat diet of high-altitude animals may potentially provide a preliminary reverse reference for the “food-flora-obesity” axis (Zhang A. et al., 2022). Chronic consumption of a HFD elevates the Firmicutes/Bacteroidetes (F/B) ratio, diminishes the abundance of beneficial bacteria such as *Bifidobacterium* and *Lactobacillus*, promotes the overgrowth of potential pathogens, and ultimately disrupts gut microbial homeostasis—an adverse shift that is avoided by high-altitude-adapted animals through their natural intake of a low-fat, high-fiber diet (Cao et al., 2019). Their gut microbiota may exhibit optimized F/B ratios, higher abundances of *Bacteroides* and *Bifidobacterium* (Chen Q. et al., 2025; Magne et al., 2020; Wang et al., 2022). In addition, the imbalance of gut microbiota in obese individuals can aggravate the risk of related metabolic diseases by enhancing energy extraction, destroying intestinal barrier, inducing chronic inflammation, interfering with endocrine signals and is involved in modulating the expression of host genes associated with lipid metabolism processes (Burcelin, 2017). It is worth noting that HFD-induced obesity and obesity caused by other conditions, such as genetic defective obesity represented by ob/ob mice, both have the common characteristics of reduced gut microbiota diversity, but the changes of HFD-induced obesity flora are reversible. However, HFD-induced dysbiosis is reversible upon dietary normalization, whereas the microbial alterations in ob/ob mice are irreversible—likely reflecting distinct underlying mechanisms. High-altitude animals rely on the steady state of flora maintained by low-fat diet, which weakens the causal chain from the source, and provides a reference for the realization of natural obesity prevention.

### 2.2 Mechanism of obesity caused by gut microbiota imbalance

Imbalance of intestinal microorganisms is an important factor leading to obesity (Koliada et al., 2017). The imbalance of gut microbiota enhances the host’s intake of dietary energy, damages the intestinal barrier function, and then induces systemic chronic low-grade inflammation, leading to the destruction of energy metabolism homeostasis (Bakker et al., 2015; Hill et al., 2012). Numerous studies have confirmed that when fecal microbiota from obese donors is transplanted into sterile recipient mice, it induces a significant increase in the recipients’ body weight and fat accumulation; in contrast, transplantation of microbial communities from lean donors attenuates the obese phenotype. This underscores that the specific assemblage of the gut microbiota is adequate to regulate the host’s energy storage capacity (Bäckhed et al., 2004). It systematically reviewed obesity-related signaling pathways, encompassing appetite regulation, adipose tissue function and energy expenditure (Wen et al., 2022). The destruction of intestinal microbiota can directly affect the energy balance of the host through metabolic pathways. Animal studies on obese individuals provide strong evidence (Jia et al., 2021; Ridaura et al., 2013).

High-fat diet and associated flora disorder can destroy intestinal epithelial tight junction through multiple ways. On the one hand, dietary fat may induce changes in the expression pattern and spatial distribution of tight junction proteins, including occludin and claudin, thus activating protein kinase C (PKC) and other signaling pathways, inducing cytoskeleton rearrangement and weakening the integrity of the barrier (Usami et al., 2003). On the other hand, the increase of hydrophobic BAs induces oxidative stress, which leads to abnormal phosphorylation of tight junction protein and dissociation from the complex (Rohr et al., 2020; Stenman et al., 2012). Dysbacteriosis reduced the production of butyric acid and other beneficial bacteria, and weakened the maintenance of energy supply and barrier function of epithelial cells. After the intestinal barrier is destroyed, bacterial products like lipopolysaccharide (LPS) translocate into the bloodstream, alterations in the expression pattern and spatial distribution of tight junction proteins (occludin and claudin) induced by dietary fat can activate the Toll-like receptor (TLR) 4/CD14-nuclear factor kappa-light-chain-enhancer of activated B cells (NF-κB) signaling pathway. This triggers a chronic inflammatory state characterized by the massive release of pro-inflammatory factors, such as tumor necrosis factor alpha (TNFα) and interleukin (IL)-6. This induces insulin resistance, forming a vicious cycle of “intestinal permeability-inflammation-metabolic dysfunction” (Cao et al., 2018; Williams et al., 2013). Furthermore, the HFD directly alters the intestinal immune environment by suppressing the secretion of barrier protective factors such as IL-10 (a major anti-inflammatory cytokine), IL-17, and IL-22 (Lorén et al., 2015; Xiong et al., 2025; Zou et al., 2018). Saturated fatty acids also mimic LPS activation of receptors like TLR 4/ TLR2, thereby intensifying inflammatory signaling (Snodgrass et al., 2013).

**FIGURE 1:**
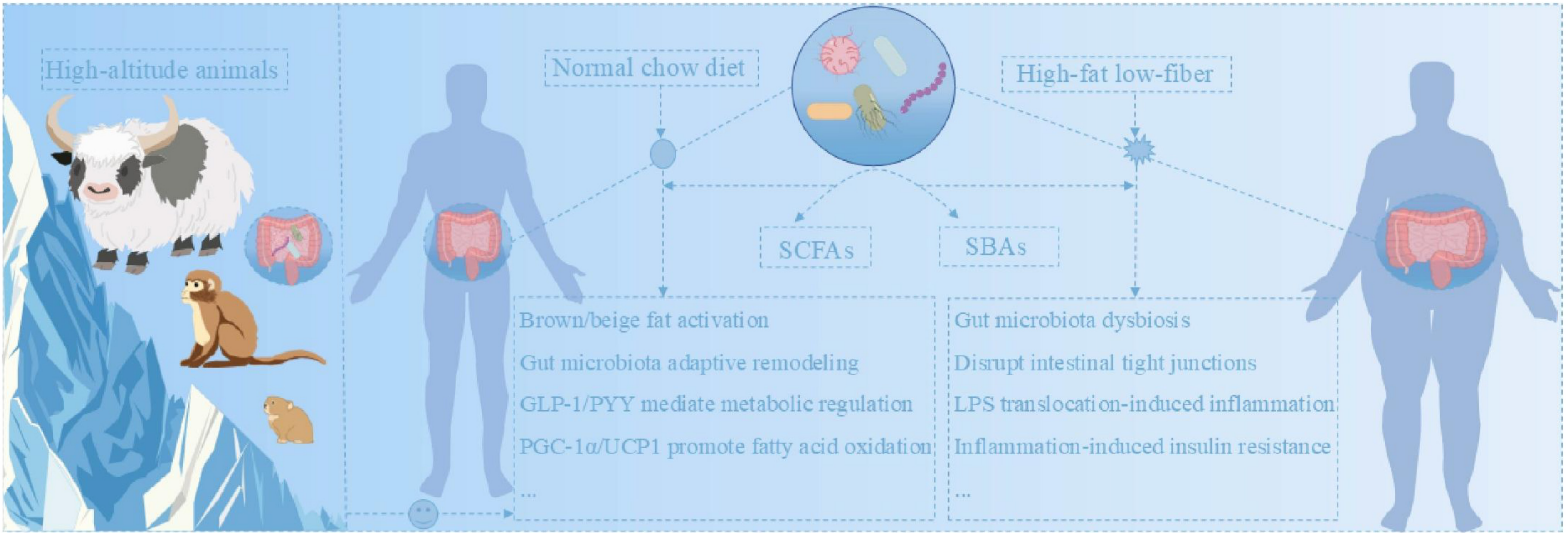
Gut microbiota function in normal versus obese states. Left: High-altitude animals: gut microbiota supports metabolic homeostasis. Middle: Normal-diet individuals: gut microbiota maintains beneficial functions. Right: Obese individuals: high-fat diet (HFD)-induced dysbiosis exerts detrimental effects.

Animals long adapted to high-altitude, low-fat environments provide a model for blocking the aforementioned obesity-driving chain through adaptive remodeling of their gut microbiota. SCFA-producing bacteria enriched in the guts of high-altitude animals reinforce the intestinal epithelial barrier by supplying substances like butyrate, helping maintain a healthy gut immune microenvironment and preventing HFD-induced disruption of tight junctions and systemic inflammation (Khoubai and Grosset, 2021). Their gut microbiota is capable of inducing intestinal L cells to secrete glucagon-like peptide-1 (GLP-1) and peptide YY (PYY), thereby precisely regulating insulin secretion and suppressing appetite. This contrasts with the hormonal dysregulation and hyperphagia caused by dysbiosis under low-altitude HFD conditions (Bautista and López-Cortés, 2025). High-altitude animals exhibit heightened brown/beige adipose tissue activity. These tissues actively take up and oxidize microbial metabolites such as succinate, rapidly reducing the concentration of succinate in the hepatic interstitial space and thereby inhibiting succinate receptor 1 (SUCNR1)-mediated hepatic inflammation (Mills et al., 2021). The microbiota and metabolites of plateau animals also stably regulate the expression of core metabolic genes in liver and adipose tissue through epigenetic mechanisms, such as peroxisome proliferator activated receptor gamma coactivator 1α (PGC-1α) and uncoupling protein 1 (UCP1) (Fernandez-Twinn et al., 2019; Wang et al., 2025). This approach ensures mitochondrial function and thermogenesis adapt to cold conditions while promoting fatty acid oxidation, effectively preventing lipid accumulation in ectopic sites. It achieves a highly efficient integration of “anti-obesity” and “energy conservation” (Figure 1).

Therefore, the distinctive adaptive traits of gut microbiota in high-altitude animals—consistent with their natural low-fat, high-fiber dietary patterns—provide compelling counterevidence for the central pathogenic role of gut microbiota dysbiosis in obesity. They also reveal a series of potential compensatory protective mechanisms, offering clues for obesity intervention strategies targeting the gut microbiome.

## 3 Mechanisms of obesity regulation: the “microbiota-metabolite-organ” interplay

### 3.1 Gut adaptation in high-altitude animals

High-altitude environment is a natural laboratory for in-depth understanding of the interaction between food, gut microbiota and host metabolism (Netzer et al., 2013). Hypoxia, low temperature and the accompanying high-fiber and low-fat diet structure together constitute the pressure of natural selection and shape the host’s intestinal micro ecosystem.

The effect of high-altitude exposure on gut microbiota has significant time-phase (Han et al., 2021). Acute exposure usually leads to dysbacteriosis and reduction of beneficial bacteria producing SCFAs (Qi et al., 2023). However, the gut microbiota of indigenous animals that have long adapted to high-altitude shows obvious evolutionary adaptation (Li and Zhao, 2015; Li et al., 2016). Similar adaptive patterns emerge across diverse taxa, including Himalayan macaques (*Macaca mulatta*) on the Tibetan Plateau (Wu et al., 2020) and other high-altitude primates, as well as plateau rodents like the Tibetan pika (*Ochotona curzoniae*) and Tibetan mole rat (*Myospalax baileyi*) (Luo et al., 2008). These animals similarly exhibit a microbial community structure dominated by the phylum Firmicutes and enriched with butyrate-producing and related metabolic functional species, thereby supporting efficient energy acquisition from high-fiber diets (Kläring et al., 2013; Li D. et al., 2023; Zhao J. et al., 2023). Among high-altitude ruminants, including yak (*Bos grunniens*) and Tibetan sheep (*Ovis aries*), gut microbes significantly enhance host energy utilization efficiency by enriching cellulose degradation and volatile fatty acid production pathways, while reducing energy loss in the form of methane (Thoetkiattikul et al., 2013). These ruminants also exhibit increased phylum-level abundance of Firmicutes, consistent with findings in primates (Ma et al., 2019). Although high-altitude birds display distinct phylum-level microbial patterns compared to mammals, they similarly show enrichment of metabolically beneficial genera such as *Lactobacillus* (Bo et al., 2022). Moreover, the prevalence of obesity in adult males at high-altitude is significantly lower (Woolcott et al., 2016). This adaptation shows cross-species convergence in microbial composition and function, that is, Firmicutes are relatively enriched. The specific functional groups related to the generation of SCFAs and the transformation of SBAs are specifically enhanced, such as the abundance and activity of butyrate-producing genera (e.g., *Pseudobutyrivibrio*) and fiber-fermenting *Prevotella* (Li et al., 2020), are significantly increased, which helps the host extract energy efficiently from low-fat and high-fiber food (Ma et al., 2021).

High-altitude adaptive animals show a significant enhancement in the production of SCFAs, particularly typified by butyric acid and propionic acid, which is closely associated with their gut microbial adaptation, which strengthened the intestinal barrier and alleviated hypoxia related inflammation, forming the basis of “low inflammation” state; SBAs are capable of enhancing tissue energy expenditure and improving insulin sensitivity by activating nuclear receptors like G protein-coupled bile acid receptor 1 (TGR5) and farnesoid X receptor (FXR) (Kim and Fang, 2018). In addition to the production of key metabolites, the gut microbiota of plateau animals also regulates the expression of genes related to nutrient transport and barrier function in intestinal epithelial cells, promotes the development of intestinal villi and increases the density of microvilli, so as to improve the absorption efficiency of nutrients and maintain the integrity of intestinal barrier. Consistent with their adaptive dietary patterns and gut microbial traits, the two metabolites (SCFAs and BAs) derived from the gut microbiota of high-altitude-adapted animals jointly achieve metabolic regulation through gut-brain, gut-liver, and other multi-organ axis networks, shaping a unique intestinal microecosystem featured by “high metabolic capacity and low inflammation.” By enriching specific functional flora, the production and function of two kinds of key metabolites, SCFAs and SBAs, are optimized, and metabolic regulation depends on the gut-X axis. This naturally formed, efficient and stable “flora metabolite organ axis” regulation mode is in contrast to HFD induced dysbacteriosis in obese individuals, providing reference for obesity intervention.

### 3.2 SCFAs as messengers regulating energy homeostasis and immune balance

Short-chain fatty acids are key messengers in the interaction between gut microbiota and host (Koh et al., 2016). Among the wide spectrum of SCFAs, acetic acid, propionic acid, and butyric acid have been the focus of intensive research, given their central roles in coordinating host immune and metabolic regulation—consistent with their previously described peripheral and central regulatory effects (De Clercq et al., 2017; Louis and Flint, 2017; Zhang et al., 1994). Butyric acid and propionic acid have anti-obesity effects and can induce the synthesis of leptin and anorexic hormone; Acetic acid may promote fat storage under certain conditions by stimulating the release of ghrelin (Crudele et al., 2023). As key signaling molecules, they regulate the host’s energy homeostasis and immune balance through multiple “gut-organ axis.” The maintenance of SCFAs level and function is crucial to metabolic health. However, the physiological functions of formic acid, valeric acid and other SCFAs need to be further explored (Ternes et al., 2022).

Research has shown that dietary supplementation with SCFAs can reduce appetite and fat accumulation by regulating relevant genes and hormones, further elucidating the potential mechanisms of SCFAs on lipid homeostasis and weight control (Jiao et al., 2021). Numerous studies have confirmed that SCFAs can either cross the blood-brain barrier or act on the central nervous system via the vagus nerve, complementing their peripheral metabolic regulatory effects, activate POMC/CART neurons in the arcuate nucleus of the hypothalamus, and promote the release of α-melanocyte-stimulating hormone (α-MSH) to inhibit appetite and increase energy consumption (Coll, 2007). Inhibition of the activity of appetite promoting neurons expressing neuropeptide Y (NPY) and agouti associated protein (AgRP) (Morton et al., 2006). Regulating the development of hypothalamic neural circuits through signal-enzyme-related protein 3 (SEMA3) and affecting energy and glucose homeostasis (Gribble and Reimann, 2019). It can activate PPARγ-PGC-1α and other signal axis, up regulate the expression of key metabolic enzymes such as uncoupling protein (UCP1/UCP2) and carnitine palmitoyltransferase I (CPT1), and promote mitochondrial thermogenesis and lipid decomposition (Den Besten et al., 2015).

As a two-way channel connecting the intestine and liver, the gut-liver axis exerts a crucial regulatory function in the coordination of lipid, energy, and overall metabolic processes (De Muynck et al., 2021; Trefts et al., 2017). Under normal circumstances, as a core metabolic organ, the liver maintains lipid homeostasis by coordinating the processes of lipid synthesis, catabolism, storage, and secretion. In obesity, excessive free fatty acids from peripheral tissues accumulate in the liver, impairing its normal lipid metabolic capacity. Consistent with their metabolic regulatory role, SCFAs can activate the deacetylase SIRT1 and its downstream PGC-1α signaling pathway in hepatocytes, which boosts fatty acid β-oxidation and represses de novo lipogenesis, thereby exerting a protective effect against hepatic lipid accumulation (Liao et al., 2024; Zhang et al., 2023). Propionic acid affects intestinal gluconeogenesis (IGN) and indirectly regulates systemic glucose homeostasis (Yoshida et al., 2019). SCFAs can stimulate intestinal L cells to secrete GLP-1 and PYY, enhance intestinal barrier function, reduce the release of inflammatory factors, and form a benign remote regulation of liver metabolism (Yi et al., 2025).

Short-chain fatty acids can regulate hematopoiesis and immune homeostasis through “gut-bone marrow axis” (Kovtonyuk et al., 2022; Lee et al., 2021). In the influenza virus infection model, SCFAs can enhance the antiviral defense ability of the host by increasing the number of Ly6c − monocytes with immune surveillance function in the circulation, so as to protect the host from pathogen infection (Trompette et al., 2018). SCFAs can also regulate the homeostasis of hematopoietic stem cells (HSCs) and affect bone marrow function in an iron metabolism dependent manner (Zhang D. et al., 2022). This regulation of hematopoiesis and immune system provides a new perspective for the effect of SCFAs on low-grade systemic inflammation, which is the core feature of obesity and metabolic syndrome.

Short-chain fatty acids constructs a metabolic regulatory network through the gut-X axis. In HFD and other pathological states, gut microbiota dysbiosis induces a marked reduction in SCFA levels, aggravating the occurrence of flora imbalance, aggravating intestinal barrier dysfunction and systemic low-grade inflammatory stress, and ultimately elevating the risk of insulin resistance and associated metabolic disorders, forming a vicious circle (Abbasi and Khodadadi, 2025; Malesza et al., 2021). Accordingly, maintaining or restoring the healthy production of SCFAs—given their multi-faceted regulatory roles in lipid metabolism, immunity, and hepatic function—serves as a key direction for the prevention and intervention of obesity and associated metabolic diseases.

### 3.3 Secondary bile acids: metabolic and immune signaling hubs in the gut-liver axis

Bile acids act both as digestive adjuvants and pivotal signaling molecules—synthesized primarily by the liver, modified by gut microbiota, and capable of targeting multiple organs to participate in systemic metabolic regulation, which expands the gut-liver-multiorgan regulatory axis previously implicated by SCFAs. They play dual regulatory roles in metabolism and immunity within the gut-liver axis. BAs are cholesterol derived steroid molecules in the liver. As cholesterol-derived steroid compounds, BA sare mainly biosynthesized in the liver, a characteristic that lays the foundation for their subsequent modification by gut microbiota and multi-organ regulatory effects. In mammals, all BAs are derived from C24-5β-cholic acid (cholic acid), whose basic functions include emulsifying dietary lipids and promoting the absorption of fat soluble vitamins (De Muynck et al., 2021).

Primary BAs synthesized by the liver enter the gut, where they are converted into SBA by specific gut microbiota, such as deoxycholic acid (DCA) and lithocholic acid (LCA) (e.g., *Clostridium* spp.) through catalysis by bile salt hydrolases and 7α-dehydroxylases (Hu et al., 2022). In obesity and other associated metabolic disorders, the balance of BAs metabolism is disrupted, usually manifested as changes in the composition of the total BAs pool, increased proportions of 12α-hydroxylated BAs, and relative enrichment of SBAs (Hori et al., 2020; Sarmiento-Andrade et al., 2022).

Disrupted BAs pools exacerbate metabolic imbalance through dual receptor mechanisms. Abnormal BAs composition may lead to tissue-specific FXR signaling abnormalities. For instance, insufficient hepatic FXR signaling suppression may promote de novo lipogenesis, while excessive intestinal FXR activation may conversely inhibit secretion of beneficial gut peptides such as glucagon-like peptide-1 (GLP-1) (Kim and Fang, 2018). The disrupted BAs pool impaired effective TGR5 activation and reduced GLP-1 secretion by intestinal L cells. This triggers uncontrolled appetite, reduces insulin sensitivity, and inhibits brown adipose tissue thermogenesis (Gou et al., 2023; Watanabe et al., 2006; Zheng et al., 2015). Specific probiotics (e.g., *Parabacteroides distasonis*) activate beneficial intestinal signaling pathways to improve metabolism by generating specific SBAs (Kuang et al., 2023).

Bile acids possess distinct immunomodulatory properties based on their hydrophobicity: hydrophobic BAs may promote inflammation, whereas hydrophilic ones often exhibit anti-inflammatory effects. Consistent with their role as gut microbial metabolites, BAs activate FXR and TGR5 to repress pro-inflammatory signaling pathways such as NF-κB, eliciting cell-type-specific anti-inflammatory responses in macrophages, hepatocytes and intestinal cells, and thus mitigating systemic inflammation to maintain metabolic homeostasis (Miyake et al., 2000). On the contrary, when inflammation occurs, inflammatory cytokines (such as TNFα and IL-1β) in turn inhibit the function of FXR, down regulate the transcription of key enzymes in the synthesis of BAs (such as CYP7A1), finely control the synthesis of BAs, form feedback regulation, and prevent cell damage and inflammation caused by its excessive accumulation (Li et al., 2012).

**FIGURE 2:**
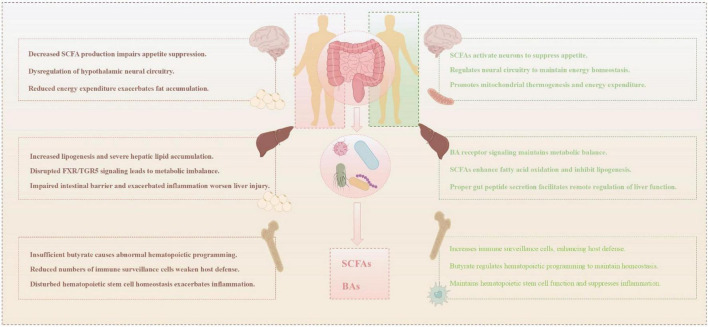
Gut-organ axes in metabolic homeostasis versus obesity. Left: Obesity-induced gut dysbiosis disrupts short-chain fatty acids (SCFAs) and bile acids (BAs) metabolism, impairing gut-brain, gut-liver, and gut-bone marrow axis signaling, which leads to appetite dysregulation, hepatic steatosis, and immune deficiency. Right: A healthy microbiota produces SCFAs and BAs that systemically coordinate energy balance, lipid metabolism, and immune homeostasis via the same axes.

Microbial transformation of SBAs and its receptor signal transduction constitute the mechanism of intestinal hepatic axis regulating metabolism and immune homeostasis. Transformation and signal blocking are important pathological links of obesity and related diseases. High-altitude animals may have evolved superior BAs metabolic homeostasis through long-term adaptation, such as maintaining beneficial SBAs profiles via specific microbiota to balance FXR/TGR5 signaling and adapt to low-fat, high-energy-expenditure metabolic demands (Chiang, 2013). Therefore, drawing from such natural adaptation models, targeting BAs metabolism through microbiome interventions (e.g., probiotics, dietary adjustments) to restore normal signaling function represents an optimal strategy for intervening in obesity and related metabolic disorders (Figure 2).

## 4 Microecological intervention strategies for obesity inspired by high-altitude adaptation

### 4.1 Dietary intervention

**FIGURE 3:**
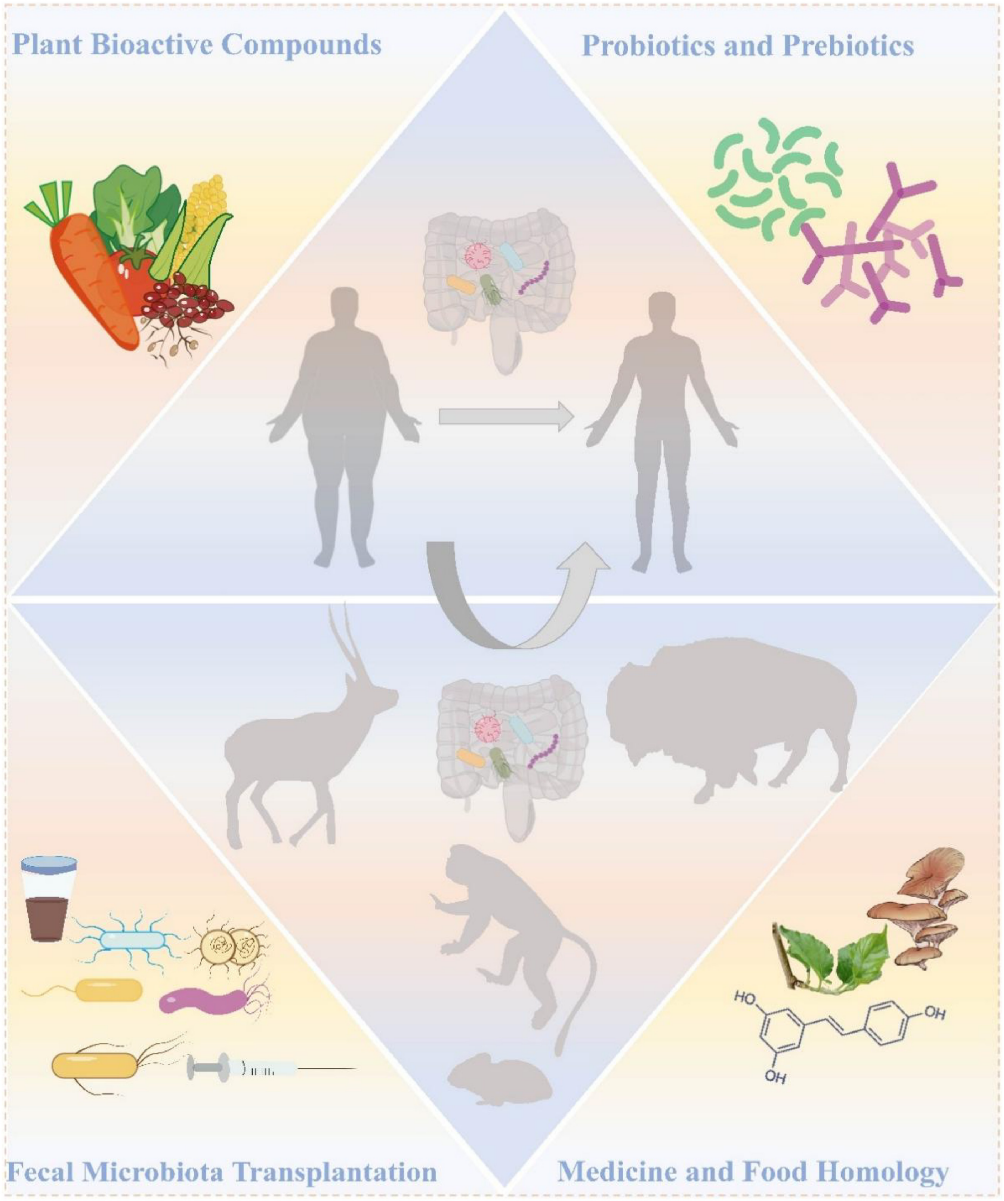
Overview of targeted interventions for the gut microbiome: prevention and treatment of obesity and related metabolic diseases. Core strategies [top left: plant-derived bioactive compounds; top right: probiotics and prebiotics; bottom left: fecal microbiota transplantation (FMT); bottom right: bioactive components based on the Medicine and Food Homology (MFH) concept].

Diet serves as the most direct determinant modulating the establishment and dynamic balance of intestinal microbiota (Figure 3). The low-fat and high-fiber diet structure of high-altitude animals is the “natural prescription” to maintain a healthy microbiota. Starting from this concept, supplementing specific plant bioactive compounds, such as dietary fiber and polyphenols, is a core strategy to mimic this beneficial environment and guide microbial regulation. Studies have shown that fruits, vegetables, grains and beans, tea and coffee, and spices are rich in plant active ingredients, which, as key substrates or regulators of microbial metabolism, stimulate the proliferation of beneficial commensal bacteria, suppress pathogenic strains, and remodel gut microbiota composition, thereby exerting anti-obesity effects (Kato, 2019; Lamichhane et al., 2022; Mir et al., 2019). Subsequently, the flora transforms plant active ingredients to produce active metabolites as a signal medium to regulate the host’s energy balance and metabolic homeostasis through the gut-X axis (Subramaniyan et al., 2025). Its anti-obesity mechanism includes activating amp activated protein kinase (AMPK) and other pathways, promoting fatty acid oxidation and thermogenesis, thus promoting the regulation of energy metabolism (Park et al., 2014). Consistent with their anti-inflammatory effects, these molecules upregulate peroxisome proliferator-activated receptor γ (PPARγ), CCAAT/enhancer-binding protein α (CEBPα), and lipid metabolism-related factors (fatty acid-binding protein 4 (FABP4), lipoprotein lipase (LPL) included), thereby inhibiting abnormal adipocyte hypertrophy and fat accumulation to maintain metabolic homeostasis (Roy et al., 2024). Inhibition of NF-κB signaling reduces levels of pro-inflammatory factors like TNF-α and IL-6, alleviating metabolic inflammation (Aruwa and Sabiu, 2024; Mitropoulou et al., 2023; Poulsen et al., 2020).

Plant components exert benefits through microbiota-mediated mechanisms. For example: White bean (*Phaseolus vulgaris*) extract reduces carbohydrate absorption by inhibiting α-amylase (Udani et al., 2004); Chlorogenic acid in green coffee and Yerba mate (*Ilex paraguariensis*) regulates lipid metabolism pathways (Gosmann et al., 2012; Li et al., 2009; Wan et al., 2013; Yang et al., 2012); Green tea (made from *Camellia sinensis* extract) activates AMPK signaling through epigallocatechin gallate (EGCG) (Rocha et al., 2016), while *Gynostemma pentaphyllum* regulate the balance between lipogenesis and lipolysis (Wang et al., 2013; Yeo et al., 2008). These effects are intrinsically linked to structural and functional remodeling of the gut microbiota, which mediates its anti-obesity potential, and show great potential in weight control, body fat reduction and improvement of glucose and lipid metabolism (Aziz et al., 2023).

Future research needs to combine multi-omics technology to explore the relationship between plant active ingredients and gut microbiota and metabolites, and provide reference ideas for the prevention and treatment of obesity induced by HFD in combination with individual differences.

### 4.2 Probiotics and prebiotics

Prebiotics and probiotics play a central role in the intervention of obesity and related metabolic diseases, and are one of the important methods to regulate intestinal microecology (Figure 3). At present, the international consensus (International Scientific Association for Probiotics and Prebiotics, ISAPP) emphasizes that prebiotics are defined as “substrates that are selectively utilized by host microorganisms and confer health benefits.” It is important to note that not all dietary fibers are prebiotics. A compound can be classified as a prebiotic in the context of gut microbiota-mediated metabolic regulation only when it selectively promotes the growth of specific gut microbial taxa and complies with rigorous requirements, namely indigestibility, gastrointestinal compatibility, targeted bacteria stimulation, and processing stability (Delcour et al., 2016; Gibson et al., 2017). The main sources of prebiotics are plant-derived oligosaccharides, such as fructans [e.g., fructose-based fructooligosaccharides (FOS), inulin] and galactans [e.g., galactose-based galactooligosaccharides (GOS)] (Lamsal, 2012; Pokusaeva et al., 2011), and starch derivatives, β-glucans, etc., (Arena et al., 2014; Zaman and Sarbini, 2016). Prebiotics are widely found in grains, fruits and vegetables (Schrezenmeir and de Vrese, 2001). In high-altitude areas, *Lactobacillus plantarum S27* has been proposed as a potential substitute for antibiotics in bird feed (Benbara et al., 2020). Probiotics are defined as “living microorganisms beneficial to the health of the host when ingested in sufficient quantities.” *Lactobacillus* and *Bifidobacterium* are the core, and can be applied only after strict safety, functionality and process feasibility screening (Gibson et al., 2017).

Probiotics and prebiotics can reshape gut microbiota homeostasis and exert anti-obesity effect. Its core function is to drive the selective expansion of beneficial bacterial taxa (*Bifidobacterium*, *Lactobacillus* included) and competitively suppress the colonization of potential pathogens, a microbial regulatory feature that aligns with the unique intestinal characteristics of high-altitude ruminants such as yak and Tibetan sheep (Geier et al., 2006; Roberts et al., 2003). In addition, probiotics and prebiotics can enhance the intestinal barrier function, reduce the release of LPS, thus blocking the chronic low-grade inflammation and endotoxemia driven by NF-κB pathway, and blocking the “intestinal leakage inflammation” cycle (Ridaura et al., 2013). Prebiotics produce SCFAs through microbial fermentation, stimulate the intestinal secretion of PYY and GLP-1, and regulate appetite and energy metabolism (Boulangé et al., 2016; Wang et al., 2019). In addition, prebiotics augment SCFA biosynthesis, upregulate the expression of genes involved in adaptive thermogenesis, and promote energy expenditure, fat oxidation, and thermogenesis—consistent with their role in shaping gut microbiota structure and maintaining metabolic homeostasis, as observed in the intestinal microecosystem of high-altitude-adapted animals (Byrne et al., 2015). In addition, probiotics can also induce mucus secretion, enhance intestinal epithelial integrity, and reduce the translocation of metabolic endotoxin, indirectly contributing to the anti-obesity effect (Slavin, 2013).

The clinical results have different benefits due to strain and individual differences (Włodarczyk and Śliżewska, 2021). The future direction should screen the next generation of probiotics of key functional bacteria of high-altitude animals and design prebiotic combinations that can specifically enrich such flora, so as to make reference for the prevention and treatment of obesity.

### 4.3 Fecal microbiota transplantation

As the cornerstone of weight management, lifestyle intervention is effective in short-term weight reduction, but its long-term weight loss outcomes are usually hard to sustain—highlighting the potential value of complementary strategies such as prebiotic intervention (Garvey et al., 2016). In addition, due to the limitations of curative effect, safety and cost, drug weight loss is restricted (Bessesen and Van Gaal, 2018). Therefore, fecal microbiota transplantation (FMT) with gut microbiota as the starting point has attracted increasing attention as a new strategy (De Groot et al., 2017; Meijnikman et al., 2018; Figure 3). Studies have indicated that FMT derived from *plateau zokors* (a plateau-endemic species) applied to low-altitude SD rats can effectively optimize pulmonary metabolism, regulate the expression profile of hypoxia-related genes, and enhance respiratory function under hypoxic conditions—supporting the significant potential of FMT in treating hypoxia-induced pulmonary hypertension, which is consistent with the adaptive gut microbial traits of plateau species (Chen Z. et al., 2025). Another study showed that transplantation of healthy lean donor flora to patients with metabolic syndrome could induce temporary metabolic improvement (De Groot et al., 2020).

Fecal microbiota transplantation has significant regulatory potential for a variety of metabolic parameters in obese patients, such as reducing caloric intake (Fetissov, 2017), fasting blood glucose (Morris, 2018), insulin resistance index (Homeostatic Model Assessment for Insulin Resistance, HOMA-IR) (Fu et al., 2022), blood pressure (Saxton et al., 2019), total cholesterol (Kaiser, 2013), and C-reactive protein (CRP) levels, etc., (Aslam, 2018). FMT can improve the structure and composition of gut microbiota to some extent, and further improve the metabolism of BAs by regulating the intestinal FXR-TGR5 signal axis (Münzker et al., 2022). On the other hand, FMT can also affect multiple pathways such as SCFAs (such as acetic acid) levels (Sanna et al., 2019), systematically regulate the host’s glucose homeostasis, lipid metabolism and inflammation, and improve HFD-induced obesity (Li Z. C. et al., 2023). The research on FMT is still continuing, and some studies have pointed out that the curative effect of FMT is heterogeneous, and the colonization of donor flora may be gradually lost, and the direct impact of FMT on anthropometric indicators such as weight and body mass index (BMI) is still lack of consistent evidence, and there may be gastrointestinal adverse reactions such as abdominal pain (De Groot et al., 2020). The direct effect of FMT on body weight is not clear, but the transplantation of characteristic flora of animals adapted to high-altitude may provide new ideas for the prevention and treatment of obesity and related metabolic diseases caused by HFD.

### 4.4 Medicine and food homology strategy

“Medicine and food homology” (MFH) originated from Chinese traditional practice, which means substances with medicinal value and nutritional function (Ng et al., 2023; Zhou et al., 2026; Figure 3). MFH substances are rich in a variety of active compounds and have diverse physiological functions. Research shows that resveratrol possesses both antioxidant and anti-inflammatory activities (Xia et al., 2017), theaflavins demonstrate antioxidant effects (Tan et al., 2019), oleic acid regulates lipid metabolism, and quercetin exhibits hypoglycemic activity (Zu et al., 2021). Additionally, a subset of MFH substances (represented by *Ganoderma lucidum*) originates from plant and fungal taxa, which are collectively referred to as plant- and fungal-derived MFHs (PMFHs) (Guo et al., 2021). It can enhance the immune system function, resist oxidative stress and inflammatory damage, and make MFH substances play an important role in regulating individual health (Zhao L. et al., 2023).

Plant- and fungal-derived medicine and food homology are the focus of research (Li et al., 2018), and the universality of their physiological mechanisms across different species has become a key issue in related studies. To date, most studies have been conducted in rodents and humans, and the core physiological mechanisms of PMFHs—such as activating the Nrf2/HO-1 antioxidant pathway (Pérez-Torres et al., 2021), suppressing TLR4/NF-κB-mediated inflammation (Li et al., 2022), modulating FXR signaling (Heni et al., 2013), regulating AMPK-driven lipid metabolism and suppressing the expression of adipogenic genes and their homologs (e.g., sterol regulatory element-binding protein-1c, SREBP1c) (Liu et al., 2024; Zhou et al., 2026)—have been shown to be largely conserved among mammals. As prebiotics, PMFHs reshape gut microbial composition by enriching SCFA-producing taxa, reducing the Firmicutes/Bacteroidetes ratio, and increasing beneficial metabolite levels (Agus et al., 2021). However, whether these mechanisms are conserved in non-mammalian spinal movements remains largely unknown. Given the significant inter species differences in baseline composition of gut microbiota, expression levels of key signaling molecules, and metabolic capacity of PMFH derived active compounds, it cannot be assumed that the PMFH mechanism is universal. The current evidence is mainly limited to mammalian models, and there is an urgent need for further research on non-mammalian species to determine the phylogenetic boundaries of PMFH biological activity.

Medicine and food homology has been recorded in ancient Chinese books, which is similar to the natural process concept of healthy flora shaped by people’s long-term diet. Therefore, the study of MFH substances and high-altitude plant species were combined to screen effective natural high-altitude plant medicinal ingredients.

## 5 Conclusion

The present review is intended to gain deeper insights into the pathogenic mechanisms underlying obesity through the analysis of the natural evolution model of “diet-flora-host metabolism” in high-altitude adaptive animal species. Continuous HFD will lead to the dysbiosis of the host gut microbiota, which will not only increase the extraction of energy by the host, but also damage the intestinal barrier, induce chronic inflammation, and make the host fat accumulate. Under the environmental selection of low-fat, high-fiber and low-oxygen, high-altitude adaptive animals form an intestinal adaptive flora characterized by “high productivity and low inflammation.” Firmicutes and related functional groups are enriched in the gut microbiota of animals adapted to high-altitude, and promote the generation of SCFAs and the transformation of SBAs, which makes obesity related flora disorder be avoided. SCFAs and SBAs act as core microbiota metabolites and signal molecules in adaptation to regulate host appetite, lipid metabolism, thermogenesis and immune balance through the “gut-X axis” network. At present, for HFD-induced obesity and metabolic diseases, there are strategies such as dietary regulation, supplementation of probiotics or prebiotics, FMT, MFH, etc., which effectively circumvent the pain points such as difficulty in adhering to traditional exercise and risks of drug treatment. Moreover, based on the research of high-altitude adaptive plants, combined with the dietary characteristics of high-altitude people, simulating the intestinal microbial ecosystem in a low-fat and high-fiber environment can further promote the synthesis of beneficial metabolites and repair the metabolic regulation association between organs. Future research needs to take the high-altitude animal intestinal adaptation model as an example, combined with multi-omics and germ-free animal models, systematically analyze the host signaling pathways triggered by the key functional strains and their characteristic metabolites of high-altitude animals, clarify the causal chain of their anti-obesity phenotype, and promote the obesity prevention and control toward a precise era.
